# Teacher mental health and workplace well-being in a global crisis: Learning from the challenges and supports identified by teachers one year into the COVID-19 pandemic in British Columbia, Canada

**DOI:** 10.1371/journal.pone.0290230

**Published:** 2023-08-31

**Authors:** Anne M. Gadermann, Monique Gagné Petteni, Tonje M. Molyneux, Michael T. Warren, Kimberly C. Thomson, Kimberly A. Schonert-Reichl, Martin Guhn, Eva Oberle

**Affiliations:** 1 Human Early Learning Partnership, School of Population and Public Health, University of British Columbia, Vancouver, BC, Canada; 2 Department of Psychology, Western Washington University, WA, United States of America; 3 Department of Psychology, University of Illinois Chicago, Chicago, IL, United States of America; McMaster University, CANADA

## Abstract

The COVID-19 pandemic and related school disruptions have led to increased concerns for the mental health of teachers. This study investigated how the challenges and systemic supports perceived by teachers during the COVID-19 pandemic were associated with their mental health and workplace well-being. This cross-sectional, survey-based study was conducted in February 2021, just prior to the third wave of the pandemic in British Columbia (BC), Canada (N = 1,276). Four multivariable linear regression models examined the associations between teachers’ pandemic-related challenges (pandemic-related personal stressors, teacher workload, difficulty implementing safety measures, meeting students’ needs), systemic supports (education system mental health and well-being support), and four mental health (psychological distress, and quality of life) and workplace well-being outcomes (job-related positive affect, turnover intentions), adjusting for sociodemographic and school characteristics. The Pratt index (*d*) was used to assess the relative importance of each predictor. A thematic qualitative analysis was conducted on teachers’ open-ended responses. Teachers’ workplace well-being (job-related positive affect and turnover intentions) was predominantly associated with their perceptions of education system support for their mental health and well-being (*d* = 46%, *d* = 41%, respectively). The most important predictor of general mental health (psychological distress and quality of life) was the number of COVID-19 related personal stressors teachers reported (*d* = 64%, *d* = 43%, respectively). The qualitative analyses corroborated and expanded upon the quantitative findings. Understanding pandemic-related challenges and supports impacting teacher mental health and workplace well-being equips us to make evidence-informed policy decisions to support teachers now and in future school disruptions.

## Introduction

There is a long-standing recognition that teaching is a stressful profession [1,2], with high levels of burnout [3] and attrition rates of up to 30% [4]. The sudden and dramatic changes introduced during the pandemic created many challenges for teachers and exacerbated already-stressful job conditions [5]. The existing research on teacher mental health during the first year of the pandemic has confirmed heightened levels of stress and emotional exhaustion among teachers in Canada [6–8] and in other countries [9–12]. A systematic review of studies conducted in 2020/2021 in China, Brazil, the United States, India, and Spain found anxiety, depression, and stress to be highly prevalent among teachers during the pandemic [13]. Although the long-term effects of the pandemic on teachers’ mental health are currently unknown, preliminary research suggests that mental health challenges persisted over the course of the pandemic. For example, a longitudinal qualitative trajectory analysis exploring teachers’ mental health at three time points in 2020 found consistent declines in teachers’ mental health [14]. In the Spring of 2021 (over 1 year into the pandemic), an empirical study measuring the mental health of Canadian school staff found significantly higher rates of anxiety symptoms compared to a representative sample of Canadian adults [15]. Similarly, teachers in the United States surveyed between the Fall of 2020 and the Spring of 2021 reported significantly higher rates of anxiety and depressive symptoms compared to respondents in other professions [16].

Over and above the general stressors experienced by individuals during the pandemic, teachers have reported a number of work-related stressors related to health and safety. As some have noted, with daily exposure to unvaccinated children and youth, teachers’ work placed them on the front lines of the pandemic but without the protections afforded other essential workers, such as earlier access to vaccines [17]. About half of teachers surveyed in the fall of 2021 in BC, Canada (where schools remained open although other social gathering restrictions were in place at this time), reported that they did not feel safe at work, and over half felt school sanitation efforts were not adequate for reducing the spread of the coronavirus [6]. The persistent uncertainty due to rapid changes in public health orders and teaching conditions (i.e., online, hybrid, and in-person) further contributed to increased stress [18]. Related to the pandemic and the associated school disruptions, teachers also reported other stressors, such as a greater need for communication and learning support from families, the need to learn new technologies, and overworking to meet the needs of vulnerable students [7,19].

Alongside the multiple challenges and stressors, it is important from a strength-based perspective to consider the supports experienced by teachers during the pandemic. Research early in the pandemic identified relationships as especially important for teachers in the United Kingdom [20]. This finding echoes previous research identifying social supports as central to the reduction of acute teacher stress [12]. Other studies conducted during the first 6 months of the pandemic suggested that school- and district-based leadership may be essential for promoting teacher well-being and preventing burnout [7,21,22]. Research conducted in this space during the COVID-19 pandemic is limited but points to the importance of support and suggests that systemic structures and supports within education systems may play a critical role in promoting positive mental health and workplace well-being during stressful periods such as the COVID-19 pandemic.

Overall, research conducted since the onset of the COVID-19 pandemic has indicated that the mental health of teachers has been compromised. However, this research predominantly focused on a limited set of mental health factors (namely, stress and anxiety), which represent only an aspect of mental health as it is generally conceptualized today. The WHO has defined mental health as “a state of mental well-being that enables people to cope with the stresses of life, realize their abilities, learn well and work well, and contribute to their community”[23]. To date, few studies examining the impact of the pandemic on teacher mental health have captured a broader definition of mental health. There is a need for more research that examines teacher mental health from a strength-based perspective (i.e., considering positive as well as negative outcomes) and that accounts for a critical, context-specific component of the overall mental health of teachers: workplace well-being (“working well”, as described in the WHO definition of mental health; [23]). Additionally, there has been limited research that has captured teachers’ own voices and focused on how challenges (i.e., any factor that caused stressed or worry in the context of the COVID-19 pandemic) and supports (i.e., perceptions of education system support related to mental health and well-being) were associated with teacher mental health and workplace well-being during the pandemic. Understanding the factors that promoted positive mental health outcomes for teachers and protected against the negative outcomes during the COVID-19 pandemic is critical from a future-facing standpoint: It provides important information on how teachers can be supported in the recovery from the pandemic and provides an opportunity to take early action to support the mental health and workplace well-being of teachers in future global crises that lead to school-related disruptions, such as a pandemic or another serious global problem impacting the world.

### The current study

The current study took a strength-based, contextualized perspective and investigated the specific challenges and supports reported by teachers during the COVID-19 pandemic and how they were associated with teachers’ mental health and workplace well-being in BC, Canada. The objective of the study was to investigate how pandemic-related challenges (e.g., workload changes, stressors, difficulty in implementing safety measures at school) and systemic supports (from colleagues, school-based administrators, school board, the BC Ministry of Education and Child Care, and union) were associated with teachers’ mental health (quality of life, psychological distress) and workplace well-being (job-related positive affect, turnover intentions) during the COVID-19 pandemic.

## Method

### Procedure

The cross-sectional, survey-based study was conducted in partnership with the BC Ministry of Education and Child Care (MOECC), and in collaboration with the BC Teachers’ Federation (BCTF). The survey was conducted online from February 3–14, 2021, at the end of the first year of the COVID-19 pandemic and just prior to the third wave when nearly 3000 cases per week (cumulative incidence of approx. 58 per 100K) were reported in BC [24]. Email survey invitations were distributed by the BCTF to a random sample of 7,000 BCTF actively employed members who work in public schools across the province. BCTF members include classroom teachers, teachers teaching on call (TTOC), adult/continuing education teachers, distributed-learning (DL) teachers, district coordinators or district helping teachers, local officers or local executive officers, special education teachers, learning assistance teachers, teacher librarians, counsellors, English language learning teachers, and Aboriginal/Indigenous Culture teachers. Prior to the survey launch, an information letter on the survey and project was sent out to all members through the BCTF newsletter. The letter and the survey explained the principles of informed consent and teachers were asked to provide their written consent on the survey at the time of survey completion. Ethics approval for this project was obtained from the University of British Columbia Behavioural Research Ethics Board.

At the time of data collection, BC schools were operating under the guidelines of the Provincial Health Officer [25] that mandated several infection prevention and exposure control measures to stop or limit transmission of COVID-19 within schools. These included avoiding activities with close face-to-face contact, assigning staff to a specific student cohort, limiting school gatherings including school assemblies and extracurricular activities, cancelling inter-school events, and mandating mask wearing indoors for all K-12 staff and middle/secondary school students (by personal or family/caregiver’s choice for elementary school students). School districts could also offer flexible in-person and remote learning options for students and families [26] and BC was one of the few contexts where in-person teaching continued uninterrupted for the 2020/2021 school year. At the time of data collection, a number of province-wide public health measures had been in place for several months, including mask mandates for indoor public settings and a restriction on social gatherings beyond household bubbles [27].

### Participants

The sample included 1,276 teacher survey respondents from across BC (a response rate of 17%). The majority of respondents identified as women (83%) and between the ages of 35 and 54 (58%). On average, respondents reported just over 15 years of teaching experience. Most of the respondents were classroom teachers (64%); the remainder were in other roles, such as special education teachers and counsellors. Most respondents reported holding a full-time teaching position (83%). Just over half of the respondents (56%) were elementary school teachers, with secondary school teachers comprising the second largest proportion (30%). Respondents were mainly from large school districts (68%). A full overview of the sample demographics is provided in Table 1.

**Table 1:** Characteristics of the analytic sample of teachers in British Columbia, Canada (*N* = 1,276).

Gender (No. (valid %))	
Women	953 (83.20)
Men	193 (16.80)
Non-binary, two-spirit, or gender identify in another way^a^	<1%
**Age (in years) (No. (valid %))**	
24 years or younger	18 (1.56)
25–34 years	232 (20.16)
35–44 years	343 (29.80)
45–54 years	328 (28.50)
55–64 years	205 (17.81)
65+ years	25 (2.17)
**Lives alone (No. (valid %))**	159 (12.50)
**Lives with children (< 18 yrs old) (No. (valid %))**	491 (38.50)
**Teaching experience (in years)**	
Mean years (*SD*)	15.54 (9.71)
Range (in years)	0–49
**Teaching position (No. (valid %))**	
Classroom teacher	776 (64.13)
Special education teacher	71 (5.87)
Teacher teaching on call (TTOC)	90 (7.44)
Learning assistance teacher	52 (4.30)
Adult/continuing education	88 (7.27)
Other^b^	133 (10.99)
**Current teaching assignment (No. (valid %))**	
Full-time	980 (82.63)
Part-time	206 (17.37)
**School type (No. (valid %))** ^**c**^	
Elementary school	708 (55.50)
Middle school	148 (11.60)
Secondary school	385 (30.20)
Other	78 (6.10)
**District size (No. (valid %))**	
Small (<200 grades 4s)	49 (4.09)
Medium (200–499 grade 4s)	158 (13.18)
Medium-Large (500–999 grade 4s)	181 (15.09)
Large (> = 1000 grade 4s)	811 (67.64)

Note. Frequencies do not always total to the full sample size (1276) due to missing data.

^a^ Masked due to small cell size.

^b^ Other includes teacher-librarians, counsellors, Distributed Learning (DL) teachers, English Language Learning teachers, among others.

^c^ Frequencies and percentages total to more than full sample (more than 100%) because respondents were able to select multiple options.

### Measures

#### Mental health and workplace well-being

We measured four outcomes of teachers’ mental health and workplace well-being: Two general indicators of mental health (quality of life, psychological distress) and two indicators of workplace well-being (job-related positive affect, turnover intentions).

*Quality of life* was assessed using a previously validated item from the PROMIS Global 10 Generic Health Questionnaire. Participants were asked, “In general, would you say your quality of life is…” with response options ranging from 1 (Poor) to 5 (Excellent) [28].

*Psychological distress* was measured using the Kessler-6 item Psychological Distress Scale (K6) [29,30]. The K6 asks respondents to indicate how often they have felt six different emotional states during the past 30 days on a scale from 0 (none of the time), 1 (a little of the time), 2 (some of the time), 3 (most of the time) to 4 (all the time). Emotional states included “…so sad nothing could cheer you up,” “nervous,” “restless or fidgety,” “hopeless,” “that everything was an effort,” and “worthless.” Previous validation research has suggested summed scores ≥ 13 indicate serious mental distress and scores ≥ 5 indicate moderate mental distress [29,31]. The scale demonstrated good internal consistency (α = 0.85).

*Job-related positive affect* was measured using two items from the *Job-related Affective Well-being Scale* (JAWS) [32] plus a third item developed for the purpose of this study by the research team. Participants were asked, “How frequently did you experience each feeling or emotion over the past 30 days?” using response options ranging from 1 (never) to 5 (extremely often or always). The following three affective states were selected based on their relevance and representation of positive job-related affective well-being for teachers in the context of the pandemic, “My job made me feel… (1) inspired; (2) satisfied; (3) a sense of purpose.” The job-related positive affect items demonstrated good internal consistency (α = 0.88).

*Turnover intentions* were measured using one item adapted from the University College London COVID-19 teacher well-being survey [33]. Participants were asked, “Has the experience of the COVID-19 pandemic made it more or less likely that you will seek to leave the profession altogether in the next few years? (please select one).” Response options were, “Yes I’m now less likely to seek to leave the profession” (scored as 1), “No I’m no more or less likely to seek to leave the profession due to COVID-19” (scored as 2), and “Yes I’m now more likely to seek to leave the profession,” (scored as 3).

#### Pandemic-related challenges and supports

**Fig 1:**
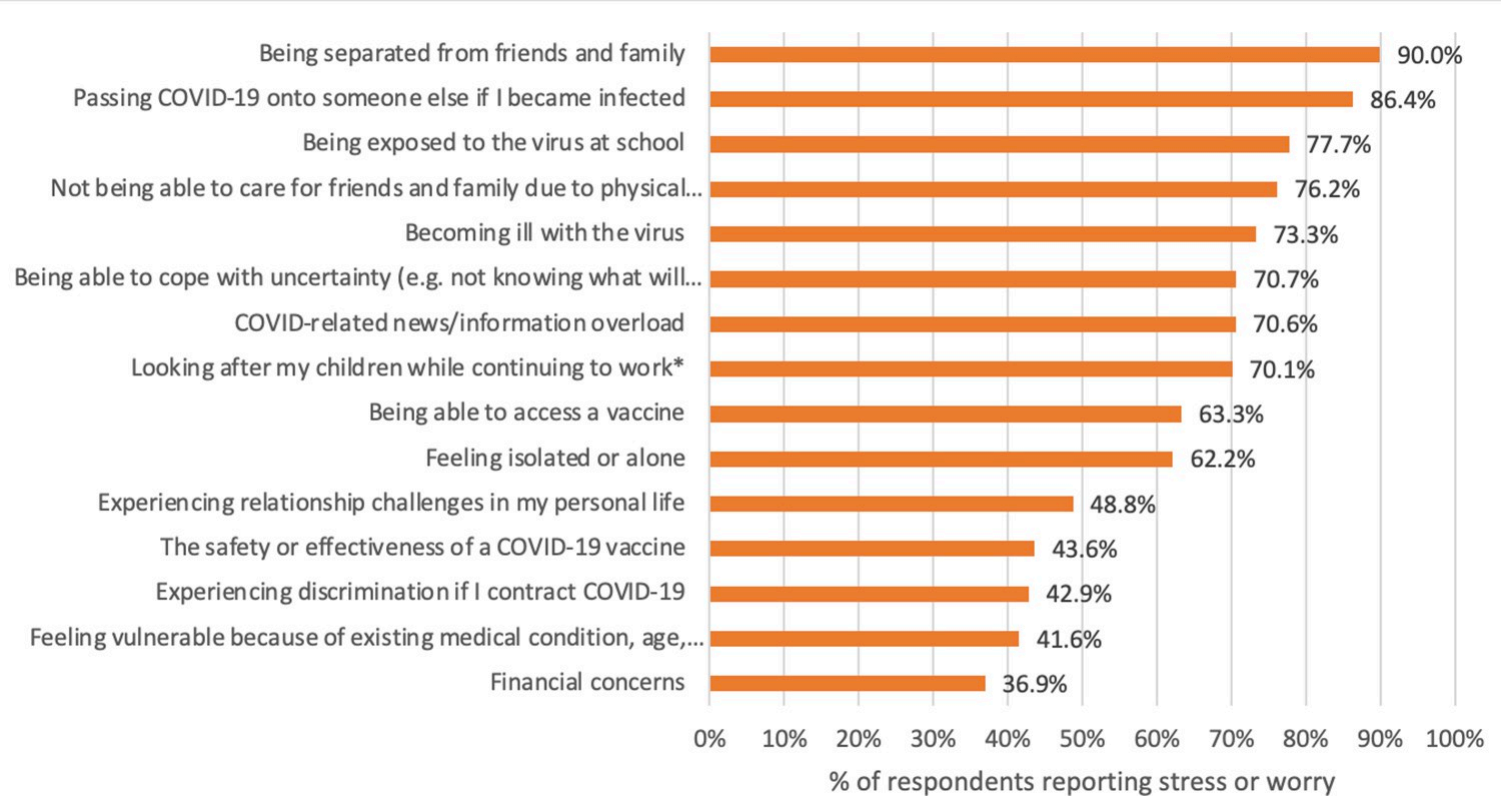
Percent of teachers who reported being stressed or worried in the past two weeks about 15 different pandemic-related personal stressors. Teachers who indicated feeling moderately, very much, or completely stressed or worried in the past two weeks about a given personal stressor were included as reporting stress or worry in that area. *Note. Only valid cases (i.e., teachers with children) were included to calculate the percentage of teachers stressed or worried about ‘looking after my children while continuing to work’.

*Pandemic-related personal stressors* were assessed using the item, “In the context of the COVID-19 pandemic, to what extent have you been stressed or worried about any of the following in the past 2 weeks?” Example stressors included “Being exposed to the virus at school” and “Feeling isolated or alone” (see Fig 1 for all items). Responses ranged from 1 (Not at all) to 5 (Completely). Binary (0/1) variables were created for each of the 15 stressors, whereby individuals who reported being ‘Moderately’, ‘Very Much’, or ‘Completely’ stressed or worried were considered to have experienced the stressor (scored as 1; lower values were scored 0). For the analysis, a composite pandemic-related personal stressor variable was created that was the sum of all 15 aforementioned binary stressor scores (with higher scores indicating exposure to more stressors; scores ranged from 0–15). This variable was adapted from a pandemic impact survey developed by members of the study team in collaboration with the Canadian Mental Health Association and UK Mental Health Foundation [34].

*Pandemic-related changes to teacher workload* was assessed using the item, “Compared to before the COVID-19 pandemic, my workload is…” Responses could range from 1 (A lot less than before) to 5 (A lot more than before). This item was adapted from a pandemic impact survey developed by the BCTF.

*Difficulty implementing safety measures* was assessed by the item, “How easy or hard is it to implement the following safety measures at your school?” Teachers were asked to respond in relation to five specific school safety measures: Wearing a mask, practicing physical distancing, washing hands (or using hand sanitizer) more often, staying home when sick, and avoiding large gatherings. Responses ranged from 1 (Very easy) to 5 (Very hard). For the analysis, a composite variable was created that represented the mean score for all five safety measures. This item was adapted from a pandemic impact survey developed by the BC Centre for Disease Control [25].

*Meeting students’ needs* was assessed using three items, “In general, during this school year, to what extent do you feel that students’ academic needs/social and emotional needs/special needs] are being met?” Responses ranged from 1 (Not at all met) to 5 (Completely met). For the analyses, a mean score of all three items was created. These items were developed by the study team in collaboration with the MOECC and BCTF.

*Education system mental health and well-being support* during the pandemic was assessed by asking teachers about the extent to which they felt supported by principals and school-based administrators, their school board, the MOECC, their union, and their colleagues. Teachers responded to the following item for each of the five potential sources of support, “In supporting your mental health and well-being during the pandemic this school year, to what extent are the following true for you?” (e.g., “I feel supported by my school board”). Responses ranged from 1 (Not at all true) to 5 (Completely true). For the analysis, a composite education system support score was calculated which represented the mean score across all five sources of support. This item was adapted from a pandemic impact survey developed by and in consultation with the BCTF.

#### School and teacher characteristics

Teachers reported the gender they most identified with (women coded as 1, men as 0 for inclusion in analyses given the small sample size for other gender identification) and their age (see age groupings in Table 1). Teachers were asked about their living situations, resulting in a ‘lives alone’ binary variable (1 = lives alone, 0 = does not live alone) and a ‘lives with children’ binary variable (1 = lives with children < 18 years old, 0 = does not). Teacher experience was assessed as the number of years of teaching experience. Respondents were asked to include experience teaching in all jurisdictions, including outside of Canada.

Teachers were asked to specify the type of teaching position(s) they held (e.g., classroom teacher or learning assistance teacher). They also indicated whether their teaching assignment was a full-time or part-time position. School type was assessed by the question, “Where do you currently teach?” Teachers could select all that apply. In BC, elementary school typically covers the years from Kindergarten to Grade 7 (ages 5–12). Secondary school covers the years from Grade 8 to 12 (ages 13–17). Some school districts have Middle school, which covers Grades 6–8. District sizes were calculated based upon the number of Grade 4 students in a given district (school districts with greater than or equal to 1000 Grade 4 students were considered large; see Table 1 for details).

#### Open-ended responses

Teachers were asked two open-ended questions: “During the pandemic this school year, if there were any structures, supports, or resources within the education system that had a positive impact on your mental health and well-being, please describe them below,” and, “If there is anything else you would like us to know about your experiences during the COVID-19 pandemic this school year, please share below.”

### Analyses

#### Quantitative analyses

Analyses were conducted using IBM SPSS software (Version 28.0). Descriptive statistics were calculated for all variables. Four multivariable linear regression models were conducted to examine the associations between teachers’ pandemic-related challenges (pandemic-related personal stressors, teacher workload, difficulty implementing safety measures, meeting students’ needs) and supports (education system mental health and well-being support) and the four mental health and workplace well-being outcomes of interest (quality of life, psychological distress, job-related positive affect, turnover intentions). All four models were adjusted for school and teacher characteristics (gender, living alone, living with children, years of teaching experience, and school type). The PRATT-index [35] characterized the relative importance of each predictor in relation to the total variance explained in each model, with values ranging from 0 (predictor accounts for none of the model’s explained variance) to 1 (predictor accounts for all of the model’s explained variance). Values below .04 were considered unimportant based upon the number of predictors included in the models in the current study [36]. We conducted complete case analyses whereby missing data were excluded listwise.

#### Open-ended survey item qualitative analysis

Utilizing NVivo 12 software (QSR International Pty Ltd, Melbourne, Australia), a six-phase thematic analysis was undertaken [37] for each open-ended survey item. For the first item (teachers were asked to describe any structures, supports, or resources within the education system that had a positive impact on their mental health and well-being), content that mapped onto the prompt to describe supports, structures, and resources were entered as free nodes and used to code the responses wherever references to them occurred. Through further discussion and analysis, these codes were developed into themes along with sub-codes that reflected our interpretation of the data. For the second item (teachers were asked to share anything else they wanted us to know about their experiences during the COVID-19 pandemic that school year), a similar process was used; however, since this item had no categories in the prompt, a fully inductive approach was utilized to generate codes and construct themes.

## Results

### Teachers’ mental health and workplace well-being

Teachers reported that their quality of life was on average ‘good’ (a midpoint scale score of 3.01, *SD* = 1.05). Of the sample, 7% reported that their quality of life was poor and 9% indicated that their quality of life was excellent. Teachers’ average summed score on the Kessler-6 item Psychological Distress Scale was 8.80 (*SD* = 4.85), indicating moderate levels of mental distress. The majority of scores were in the moderate mental distress stress range (56%), 23% of respondents’ scores were in the ‘serious mental distress’ range and another 21% indicated typical levels of mental distress. On the Job-related Affective Well-being Scale, the average teacher score was 3.05 (*SD* = .80) which corresponds to ‘sometimes’ their job made them feel positive affect (e.g., inspired, satisfied) during the past 30 days, with 24% of the sample indicating never or rarely and 29% indicating quite often, extremely often or always feeling job-related positive affect. On average, teachers reported that they were between ‘no more or less likely’ to ‘more likely’ to seek to leave their profession in the next few years due to the experience with the COVID-19 pandemic (representing a score of 2.39 (*SD* = .52) on the scale). Broken down, 41% reported that they were now more likely to seek to leave the profession and 2% indicated that they were now less likely to leave the profession due to the experience of the COVID-19 pandemic.

### Teachers’ challenges and supports

**Fig 2:**
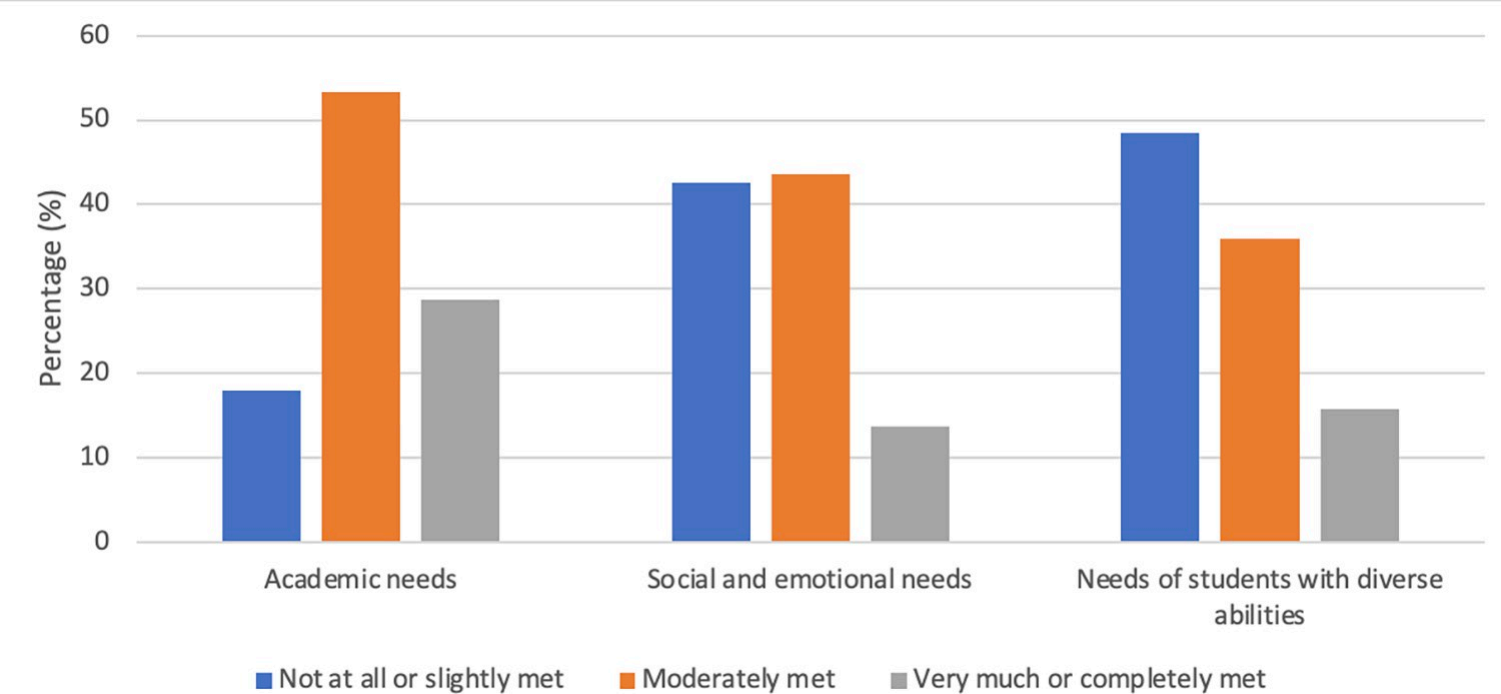
Teachers’ responses (percentage breakdown) about the extent schools were meeting students’ academic needs, social and emotional needs, and the needs of students with diverse abilities this school year.

Table 2 provides a descriptive summary of teachers’ reported school experiences during the COVID-19 pandemic. On average, teachers reported that their workload had increased compared to before the pandemic. When asked about personal stressors related to the pandemic, they reported feeling moderately to completely stressed or worried on about 9 of the 15 (SD = 3.47) potential stressors. Fig 1 lists all 15 personal stressors and the percentage of teachers who indicated being moderately to completely stressed for each. The three most commonly reported stressors were ‘being separated from friends and family’, ‘passing COVID-19 onto someone else if I became infected’, ‘being exposed to the virus at school’ (90.0%, 86.4%, and 77.7% of teachers reported this stressor, respectively). Teachers generally reported that implementing safety measures during the COVID-19 pandemic was ‘somewhat easy’ to ‘neither hard nor easy’, with the exception of ‘practicing physical distancing’, which respondents generally reported was ‘somewhat hard’ to ‘very hard’ (see Table 2). Mean scores presented in Table 2 indicate that, on average, teachers reported feeling that students’ academic needs were ‘somewhat met’ during the school year but that students’ social and emotional needs and the needs of students with diverse abilities were only ‘slightly met’ to ‘moderately met’ (see Fig 2 for response breakdowns in percentages). When asked about sources of mental health and well-being support in the education system during the pandemic, teachers reported feeling the most support from colleagues, followed by principals and school-based administrators, and the least support from the Ministry of Education and Child Care (see Table 2).

**Table 2:** Descriptive measures of teachers’ reported school experiences during the COVID-19 pandemic.

Pandemic-related workload changes (*M(SD*))^a^	3.80 (0.92)
**Number of pandemic-related worries or stressors (*M(SD*))** ^**b**^	9.03 (3.47)
**Difficulty implementing COVID-19 safety measures at school (*M(SD*))** ^**c**^	3.07 (0.96)
Wearing a mask	2.82 (1.47)
Practicing physical distancing	4.22 (1.13)
Washing hands [or use hand sanitizer] more often	2.51 (1.31)
Staying home when sick	2.81 (1.35)
Avoiding large gatherings	3.00 (1.42)
**Student needs met (overall) (*M(SD*))** ^**d**^	2.82 (0.66)
Meeting students’ academic needs	3.13 (0.78)
Meeting students’ social and emotional needs	2.67 (0.81)
Meeting the needs of students with diverse abilities	2.61 (0.89)
**Education system mental health and well-being support (overall) (*M(SD*))** ^**e**^	2.86 (0.77)
Principal and school-based administrators	3.27 (1.24)
School board	2.25 (1.09)
Ministry of Education and Child Care	1.81 (0.95)
Union	3.15 (1.14)
Colleagues	3.79 (0.93)

^a^ Compared to before the COVID-19 pandemic, workload changes reported on a scale from 1(*A lot less than before*) to 5 (*A lot more than before*).

^b^ Number of worries or stressors in the past two weeks in the context of COVID-19. All items where respondents indicated they were ‘Moderately’ to ‘Completely’ stressed or worried were included in the sum score (up to 15 stressors possible; range was 0–15).

^c^ On a scale from 1 (*Very easy*) to 5 (*Very hard*).

^d^ The extent teachers felt students’ needs were being met this school year, on a scale from 1 (*Not at all met*) to 5 (*Completely met*).

^e^ The extent teachers felt mental health and well-being were supported during the pandemic this school year, on a scale from 1 (*Not at all true*) to 5 (*Completely true*).

### Predictors of general mental health for teachers

Table 3 presents unstandardized regression model coefficients, confidence intervals and PRATT-index values for models predicting quality of life and psychological distress. Statistically significant predictors that met the PRATT-index cut-off criterion of relative importance (i.e., 0.04) are presented here. The relatively most important predictor of both quality of life (negatively predictive) and psychological distress (positively predictive) was the number of personal stressors related to the COVID-19 pandemic (*d* = 43% and 64%, respectively). This was followed in importance by COVID-19-related increased workload, which was negatively predictive of quality of life (*d* = 16%) and positively predictive of psychological distress (*d* = 18%). Perceptions of education system supports for mental health and well-being were positively predictive of quality of life (*d* = 16%) and negatively predictive of psychological distress (*d* = 7%). Perceptions that students’ needs were being met during the school year were positively related to quality of life (*d* = 9%) and negatively related to psychological distress (*d* = 6%).

**Table 3:** Regression models predicting quality of life and psychological distress amongst a sample of BC teachers during the COVID-19 pandemic.

	Quality of Life			Psychological Distress [sum score]		
	***B (SE)***	***CI (95%)***	***Pratt Index*** ^***a***^	***B (SE)***	***CI (95%)***	***Pratt Index*** ^***a***^
**Gender**						
Men	Reference	Reference	Reference	Reference	Reference	Reference
Women	-0.02 (.08)	-0.19,0.14	<0.01	0.12 (.33)	-0.52,0.76	<0.01
**School Type**						
Elem	Reference	Reference	Reference	Reference	Reference	Reference
Middle	-0.13 (.09)	-0.31,0.05	<0.01	0.04 (.36)	-0.68,0.75	<0.01
Second	-0.04 (.07)	-0.17,0.09	<0.01	0.17 (.26)	-0.34,0.68	<0.01
Other	-0.16 (.13)	-0.41,0.09	0.01	-0.53 (.51)	-1.53,0.47	<0.01
**Teach Exp.**	-0.05 (.03)	-0.11,0.01	<0.01	-0.51 (.12)	-0.74,-0.28	0.04
**Live alone**	-0.44 (.09)***	-0.62,-0.26	0.14	1.05 (.36)**	0.34,1.75	0.02
**Live w/ children**	0.07 (.06)	-0.05,0.20	0.02	-0.54 (.25)*	-1.02,-0.05	0.01
**COVID-19 Workload**	-0.13 (.03)***	-0.19,-0.06	0.16	1.01 (.12)***	0.77,1.25	0.18
**COVID-19 Stressors**	-0.22 (.03)***	-0.28,-0.16	0.43	2.17 (.13)***	1.91,2.42	0.64
**Difficulty w/ safety meas.**	-0.06 (.03)	-0.12,0.01	0.06	0.11 (.13)	-0.15,0.36	0.01
**Student needs**	0.08 (.03)**	0.02,0.14	0.09	-0.44 (.12)***	-0.68,-0.20	0.06
**COVID-19 education system supports**	0.10 (.03)**	0.04,0.17	0.16	-0.36 (.13)**	-0.62,-0.10	0.07

R^2^_adj [quality of life]_ = 0.152; R^2^_adj [psychological distress]_ = 0.382.

Missing data were excluded listwise for both quality of life and psychological distress models (n = 1198, n = 1191, respectively).

Statistically significant values are indicated by asterisk (* = < .05

** = < .01

*** = < .001).

^a^ Pratt Index values smaller 0.04 indicate predictors that are relatively unimportant in explaining the model variance.

### Predictors of workplace well-being for teachers

Table 4 presents regression model coefficients, confidence intervals and PRATT-index values for models predicting both job-related positive affect and turnover intentions. Statistically significant predictors that met the PRATT-index cut-off criterion of relative importance (i.e., 0.04) are presented here. Teachers’ perceptions of support for their mental health and well-being was the relatively most important predictor of workplace well-being. Perceptions of support was positively associated with job-related positive affect (*d* = 46%) and negatively associated with turnover intentions (*d* = 41%). Perceptions that students’ needs were being met during the school year was a relatively important positive predictor of job-related positive affect (*d* = 23%) and to a lesser degree, a negative predictor of turnover intentions (*d* = 6%). Teachers’ reported number of personal stressors related to the COVID-19 pandemic was also a relatively important predictor of job-related positive affect (negatively predictive; *d* = 20%) and turnover intentions (positively predictive; *d* = 22%). Difficulty with safety measures was a negative predictor of job-related positive affect (*d* = 9%) and a positive predictor of turnover intentions (*d* = 8%). Years of teaching experience was positively associated with turnover intentions (*d* = 14%), such that those who had been teaching longer had higher turnover intentions.

**Table 4:** Regression models predicting job-related positive affect and turnover intentions amongst a sample of BC teachers during the COVID-19 pandemic.

	Job-related positive affect			Turnover intentions		
	***B (SE)***	***CI (95%)***	***Pratt Index*** ^***a***^	***B (SE)***	***CI (95%)***	***Pratt Index*** ^***a***^
**Gender**						
Men	Reference	Reference	Reference	Reference	Reference	Reference
Women	0.11 (.06)	-0.01,0.23	0.01	0.02 (.04)	-0.07,0.10	<0.01
**School Type**						
Elem	Reference	Reference	Reference	Reference	Reference	Reference
Middle	0.08 (.07)	-0.06,0.21	0.01	0.02 (.05)	-0.08,0.11	<0.01
Second	-0.04 (.05)	-0.13,0.05	<0.01	-0.02 (.03)	-0.08,0.05	<0.01
Other	0.14 (.09)	-0.04,0.32	0.01	0.004 (.07)	-0.12,0.13	<0.01
**Teach Exp.**	-0.04 (.02)	-0.08,0.01	<0.01	0.08 (.02)***	0.05,0.11	0.14
**Live alone**	-0.03 (.07)	-0.16,0.10	<0.01	0.06 (.05)	-0.03,0.15	0.01
**Live w/ children**	-0.02 (.05)	-0.11,0.06	<0.01	-0.07 (.03)*	-0.13,-0.01	0.03
**COVID-19 Workload**	-0.02 (.02)	-0.07,0.02	0.02	0.04 (.02)*	0.01,0.07	0.08
**COVID-19 Stressors**	-0.12 (.02)***	-.016,-0.07	0.20	0.07 (.02)***	0.04,0.10	0.22
**Difficulty w/ safety meas.**	-0.07 (.02)***	-0.11,-0.02	0.09	0.03 (.02)	-0.002,0.06	0.08
**Student needs**	0.15 (.02)***	0.10,0.19	0.23	-0.04 (.02)*	-0.07,-0.004	0.06
**COVID-19 education system supports**	0.21 (.02)***	0.16,0.26	0.46	-0.11 (.02)***	-0.15,-0.08	0.41

R^2^_adj [positive affect]_ = 0.230; R^2^_adj [turnover intentions]_ = 0.160.

Missing data were excluded listwise for both positive affect and turnover intentions models (n = 1193, n = 1134, respectively).

Statistically significant values are indicated by asterisk (* = < .05

** = < .01

*** = < .001).

^a^ Pratt Index values smaller 0.04 indicate predictors that are relatively unimportant in explaining the model variance.

### Teachers’ open-ended responses on education system structures, supports, and resources

When invited to comment on whether any structures, supports, or resources within the education system had a positive impact on mental health and well-being, approximately one-third of respondents commented (33%; of these, 86% identified as women, with an average teaching experience of 18 years; 60% were elementary, 9% middle, and 29% secondary school teachers). Themes related to the structures, supports, and resources were identified through our qualitative analysis (see S1 Table): With respect to supports, colleagues were most frequently cited by teachers as being a key source of support that had a positive impact on mental health. As one teacher describes:

The only thing that helps me to get through the day is by talking and collaborating with the teachers that are in the classrooms beside me. Together we have formed a small group of support for each other. We look out for each other and debrief daily. If it weren’t for them, I would feel totally alone and isolated (Elementary teacher, identified as a woman, 17 years of teaching experience).

Another theme to emerge was with respect to supports from administration and the role they played by acknowledging and supporting well-being and encouraging work-life balance. One teacher wrote, “I appreciate my bosses checking in with my health and well-being often and making suggestions to help support my workload” (elementary/middle school teacher, identified as a woman, 1 year of teaching experience). Teachers also cited (although less frequently) the positive role that school board/districts, unions, and recognition within school and the community played in their mental health. In terms of structural supports, a prominent theme emerged around safety protocols. Teachers frequently expressed that the implementation of school safety protocols had a positive impact on mental health. In the words of one teacher, “offering masks and hand sanitizer make it easier for students and staff to access this bit of PPE (personal protective equipment) and this makes me feel a bit more safe” (Secondary teacher, identified as a woman, 11 years of teaching experience).

Teachers also referred to resources that had a positive impact on mental health and well-being. Three sub-themes related to resources emerged with respect to wellness initiatives, online learning, and social-emotional learning (SEL). The most prominent resource sub-theme was related to the positive role that wellness initiatives played in supporting teacher mental health. As one respondent describes, “school-based wellness and spirit committees have been very helpful in promoting feelings of staff well-being and connection” (elementary teacher, identified as a woman, 7 years of teaching experience). In contrast, some teachers did not perceive any education system supports, structures, or resources as supporting their mental health and well-being. For example, as one teacher put it, “the admin sent out emails telling us to ‘take care of yourselves’ but did nothing to take care of us” (Secondary teacher, identified as a woman, 20 years of teaching experience).

### Teachers’ open-ended responses about their experiences during the COVID-19 pandemic

When invited to share more generally about their experiences during the COVID-19 pandemic, nearly half of the sample responded (46%; 83% women, average years of teaching experience was 17 years, 60% elementary, 10% middle, 30% secondary school teachers). Four overarching themes emerged around changes in mental health, changes in professional life, concerns regarding the pandemic response, and student-related feedback (see S2 Table). With respect to changes in mental health, we identified four sub-themes related to teachers’ worries about exposure to the virus, experiencing increased stress, feeling burnt out and exhausted, and feeling frustrated. One teachers’ statement illustrates the worry about exposure experienced by many teachers:

I purchased my own plexiglass screens for my classroom and desk at my own expense approximately $460. I read the news at lunch and when it was announced that there would be no essential workers included in phase 2 of the vaccination program I sat behind my (plexiglass) screen at my desk with my students in my class eating their lunch and cried (Secondary teacher, identified as a woman, 24 years of teaching experience).

Nine different sub-themes related to changes in professional life were identified (see S2 Table for a full list). The most frequent sub-theme was related to the challenge of enforcing safety protocols. One teacher describes the challenges of maintaining physical distancing in the classroom:

It is very challenging to keep students apart. If social distancing and no touching within a cohort was truly meant to be implemented, we should have had smaller class sizes or extra staff to keep students apart. It’s very unrealistic. Young children are impulsive and need the touch. They wrestle, hug, jump on each other (Elementary teacher, identified as a woman, 1 year of teaching experience).

Two sub-themes emerged related to inconsistency in public health orders and implementation as well as feeling unsupported by the government. One teacher stated:

The lack of transparency and inconsistent implementation and interpretation of PHO (Public Health Orders) is a source of constant stress. Especially when the public and school orders do not match. I’m really upset when the statement is said that there is no data that supports school transmission when the data is not being collected or accurately collected (Middle school teacher, identified as a woman, 16 years of teaching experience).

With relation to student-related feedback, sub-themes emerged around being unable to meet students’ needs, missing connection with students and families, reduced learning time, and being glad students are in school. Of the four sub-themes, teachers most frequently raised concern about not being able to meet students’ needs. As described by one teacher:

There are far more students struggling with anxiety and mental health and we’re struggling to provide enough support which creates even more stress and anxiety for teachers because we feel we are not supporting our students to the best of our ability (Elementary teacher, identified as a woman, 10 years of teaching experience).

## Discussion

The impacts of the COVID-19 pandemic have exacerbated stressors that already existed within the teaching profession and introduced new ones [38]. The current study sought to investigate associations between pandemic-related challenges (e.g., workload changes, stressors, difficulty implementing safety measures at school) and supports (e.g., from colleagues, school-based administrators, school board, the MOECC, and union) and indicators of teacher mental health and workplace well-being (job-related positive affect, turnover intentions, psychological distress, and quality of life). Data collection occurred in the 2020–2021 school year during which in-person teaching continued uninterrupted in BC. The majority of teachers in this study met the range for ‘moderate mental distress’ (56%) and ‘serious mental distress’ (23%) using the K6 screening scale for psychological distress [29]. This is a substantially higher proportion in comparison to previous studies using this measure with teachers (moderate mental distress: 50%; serious mental distress: 13%) [39] and with the general population (moderate mental distress: 28%; serious mental distress: 9%) [31].

Chief among our findings was that teachers’ job-related positive affect and turnover intentions were primarily associated with their perceptions of support for mental health and well-being from the education system, over and above the relative importance of COVID-19 stressors and workload–a finding that was emphasized and further illustrated through the open-ended responses. This finding suggests that supports offered by educational systems can be a powerful lever for change regarding teachers’ mental health and workplace well-being. Recent research has explored the relation between supports within the educational system and teachers’ professional or occupational well-being both during and prior to the COVID-19 pandemic. For example, Alves and colleagues [2020] conducted a cross-sectional study of Portuguese teachers’ satisfaction with the education system in relation to their well-being before and during the pandemic[40]. In line with our findings, teachers reported reduced professional well-being during the pandemic, and increased turnover intentions. van Horn and colleagues [2010] identified four dimensions of teachers’ occupational well-being: cognitive, subjective, physical and mental, and social well-being[41]. The latter dimension, social well-being, emphasizes the importance of a supportive school culture in which teachers enjoy positive interactions and working relationships with administrators, colleagues, families, and students. A recent systematic review of the literature on teacher well-being found positive relationships with colleagues, students, and parents; a supportive work environment; community recognition; and support by principals/leadership to be significant and positive predictors of teacher well-being in dozens of studies [42]. Notably, the authors concluded that three groups in particular complement each other in supporting teacher well-being: “Principals can provide positive and supportive working environments; teachers can support each other, work together, moderate stress and the demands of professional life, and help with emotion regulation; and students can give meaning to the teaching profession and reward teachers’ work,” [p. 13] [42]. This is corroborated in both our quantitative and qualitative data in which supportive relationships with colleagues and feeling supported by the school community surfaced as important factors associated with teachers’ workplace well-being. Thus, whether situated in the context of a pandemic, other global crisis (i.e., one or more serious problems impacting the world), or in more “normal” times, education systems can promote teachers’ well-being by fostering strong, supportive school cultures and positive relationships amongst all stakeholders.

Teachers’ occupational well-being has been associated with the quality of their relationships with their students and, during the pandemic, these relationships were significantly challenged. Prominent among our findings was that teachers’ perceptions of their ability to meet the needs of students was an important predictor of job-related positive affect. That teachers have struggled to meet the needs of students during the pandemic has been reported elsewhere [43]. Recent research has identified relations between teachers’ self-efficacy and well-being during the pandemic [44,45]. For example, Herman and colleagues [2021] surveyed over 600 US teachers before and during the pandemic and found teachers’ perceived efficacy to be a strong predictor of well-being outcomes during the pandemic [45]. Further, Beard and colleagues [2021] noted a relation between teachers’ sense of efficacy and their ability to support students’ social and emotional needs during the pandemic [46]. Describing teachers as “first responders for students’ well-being” [p. 3], the researchers sought to understand if and how teachers’ responses to students’ social and emotional needs during the pandemic also supported their own social and emotional needs, including the feeling of efficacy. They noted that innovative approaches such as online learning communities that connected teachers in communities of practice, and social media groups that buoyed their spirits, helped teachers both learn how to better support their students’ needs in the pandemic while also promoting their own well-being. This pandemic example of how bolstering supports for teachers can simultaneously help meet students’ needs and support teachers’ well-being is instructive for current and future policy-decisions around systemic supports for teachers. Identifying support factors that buffer teacher mental health and well-being during the pandemic (e.g., social and emotional learning (SEL) training for educators and for students [46,47]) can help inform systemic changes that build on these supports to promote teachers’ well-being now and in the future.

### Limitations

There are certain study limitations to highlight. First, the study does not claim a representative sample and as such, the findings should be interpreted with caution. The survey was voluntary and the sample of participants may be biased in certain ways. For example, it is possible that teachers who chose to participate in the survey were those that had more capacity to respond and were more likely to report positive mental health and workplace well-being. Alternatively, it is possible that teachers who were particularly unhappy with their workplace circumstances were more likely to respond and more likely to report lower mental health and workplace well-being and fewer education system supports. Second, the study was not able to account for baseline mental health and workplace well-being prior to the COVID-19 pandemic. A lack of baseline hindered our ability to measure and account for differences in teachers’ mental health and workplace well-being and compare to pre-pandemic levels.

### Conclusion

This study makes important contributions to our understanding of teacher mental health and workplace well-being in the face of the COVID-19 pandemic as well as future global crises and related school disruptions and identifies key challenges and supports impacting the mental health and workplace well-being of teachers. Understanding the key challenges and supports are essential to inform much-needed mental health and workplace well-being recovery efforts for teachers now, and as part of the pandemic recovery. Beyond this, our research and other research focused on the impact of the COVID-19 pandemic on the mental health and workplace well-being of teachers is critical for future planning so that we can (1) identify the most effective ways to build up a strong system of mental health and workplace well-being support for teachers moving forward and (2) to ensure that appropriate supports are in place in the event of future pandemics or other crises that disrupt education systems around the world.

## Supporting information

S1 Table Teachers’ qualitative comments and resulting themes related to education system structures, supports, and resources that had a positive impact on mental health and well-being. (DOCX)

S2 Table Teachers’ qualitative comments and resulting themes related to their experiences during the COVID-19 pandemic. (DOCX)
