# Supplementary material for: Teacher mental health and workplace well-being in a global crisis: Learning from the challenges and supports identified by teachers one year into the COVID-19 pandemic in British Columbia, Canada
Source: PLoS One. 2023 Aug 31;18(8):e0290230. doi: 10.1371/journal.pone.0290230 (PMC10470891; doi:10.1371/journal.pone.0290230)
Supplement: S1 Table — (DOCX) [file pone.0290230.s001.docx]

**S1 Table. Teachers’ qualitative comments and resulting themes related to education system structures, supports, and resources that had a positive impact on mental health and well-being**

| **Question: During the pandemic this school year, if there were any structures, supports, or resources within the school system that had a positive impact on your mental health and wellbeing, please describe them below.** | | | | |
| --- | --- | --- | --- | --- |
| **Code** | **Sub-code** | **Frequency** | **Quote(s)** | **Teacher Demographics**  **(gender, yrs. teaching, level(s) taught)** |
| Support | Administration | 91 | - I appreciate my bosses checking in with my health and wellbeing often and making suggestions to help support my workload. - Our administrators are very appreciative of all of our efforts at this time and let us know it. They do not question if we need time to be with family members who are aged and suffering and even encourage me to take the time I need… | - F, 1, elementary - F, 40, elementary |
|  | Colleagues | 113 | - It is not the structures or resourcesm, it is my wonderful colleagues who are helping each other through this. - The only thing that helps me to get through the day is by talking and collaborating with the teachers that are in the classrooms beside me. Together we have formed a small group of support for each other. We look out for each other and debrief daily. If it weren’t for them, I would feel totally alone and isolated. | - F, 25, elementary - F, 17, elementary |
|  | Recognized w/in school | 4 | I feel that my district has been clear that they believe we are working under extremely trying circumstances and that they are trying to help us do the best we can with the given circumstances. It seems like there is a lot of trust in me (us) from the parent community and the administration in general. | F, 32, elementary |
|  | Recognized w/in community | 7 | Appreciation for us as front line workers. We put our own families at risk everyday to look after other people’s children | F, 25, secondary |
|  | School board/district | 21 | My district kept telling me that I come first and my mental health is important. This has enabled me to give myself permission to leave work a bit earlier and go for a run before I come home to taking care of my family and has helped my mental health. | F, 4, elementary |
|  | Ministry of Ed | 1 | clearer and more consistent communication from government authorities | F, 20, secondary |
|  | Union | 17 | The BCTF has done a great job staying vocal about safety concerns - we appreciate the support. | F, 16, secondary |
| Structures | Safety protocols | 118 | - I feel the school district has used money wisely to support cleanliness in our schools. Most clean of my entire career - offering masks and hand sanitizer make it easier for students and staff to access this bit of PPE and this makes me feel a bit more safe. It also encourages the use of masks versus students showing up with bandana style face coverings which I don't feel offer any protection | - F, 28, elementary - F, 11, secondary |
| Resources | Wellness initiatives | 54 | School based wellness and spirit committees have been very helpful in promoting feelings of staff wellbeing and connection. | F, 7, elementary |
|  | Online learning | 10 | Learning to effectively use some of the online applications and platforms has been beneficial in helping to stay connected and work as a team regardless of the situation. | F, 19, elementary |
|  | SEL program | 6 | In the first 6 weeks of school I had an extra prep block while a social emotional expert teacher worked with my class. | F, 35, elementary |
| None | | 23 | No. The admin sent out emails telling us to “take care of ourselves” but did nothing to take care of us. | F, 20, secondary |
| Note. Total responses = 423/1276 | | | | |
