# Supplementary material for: Teacher mental health and workplace well-being in a global crisis: Learning from the challenges and supports identified by teachers one year into the COVID-19 pandemic in British Columbia, Canada
Source: PLoS One. 2023 Aug 31;18(8):e0290230. doi: 10.1371/journal.pone.0290230 (PMC10470891; doi:10.1371/journal.pone.0290230)
Supplement: S2 Table — (DOCX) [file pone.0290230.s002.docx]

**S2 Table. Teachers’ qualitative comments and resulting themes related to their experiences during the COVID-19 pandemic**

| **Question: If there is anything else you would like us to know about your experiences during the COVID-19 pandemic this school year, please share below.** | | | | |
| --- | --- | --- | --- | --- |
| Code | **Sub-Code** | **Frequency** | **Quote(s)** | **Teacher Demographics**  **(gender, yrs. teaching, level(s) taught)** |
| Changes in mental health | Experiencing increased stress | 59 | - There are far more students struggling with anxiety and mental health and we’re struggling to provide enough support which creates even more stress and anxiety for teachers because we feel we are not supporting our students to the best of our ability. - This has been the hardest year of my teaching career. | - F, 10, elementary - F, 16, elementary |
|  | Experiencing worry about exposure | 79 | I purchased my own plexiglass screens for my classroom and desk at my own expense approximately $460. I read the news at lunch and On Friday, when it was announced that there would be no essential workers included in phase 2 of the vaccination program I sat behind my [plexiglass] screen at my desk with my students in my class eating their lunch and cried. I am a cancer survivor in the last four years and I go to work every day and because of my age I will not be vaccinated till August if I am lucky and there are no more delays. My friends outside of teaching cannot understand how I can be allowed to have 27 kids in my classroom with no physical distancing every day. | F, 24, secondary |
|  | Feeling burnt out and exhausted | 40 | - I would like the government to stop treating school staff as disposable. I have never felt more discouraged, unappreciated and deflated while at the same time burned out. The way we’ve been treated during this pandemic has really changed my outlook on the profession. I am currently looking for a change of career and so is my husband (he’s a teacher as well). - Myself and my colleagues all feel the weight of the pandemic and are exhausted. We are all noticing increased anxiety and mental health issues with our students, and stress amongst the families. The lack of support for these students weighs on us as we are all overloaded. | - F, 15, elementary - F, 25, elementary |
|  | Feeling frustrated | 26 | - I am constantly frustrated by how we as teachers are being treated by the PHO and the govt. - I have felt increasingly frustrated by the loss of academic standards in our school, but that was happening before the pandemic. This whole experience has just given more rationalization to allowing standards to slip even further. | - F, 25, elementary/secondary - M, 15, secondary |
| Changes in professional life | Challenging to enforce safety protocols | 91 | It is very challenging to keep students apart. If social distancing and no touching within a cohort was truly meant to be implemented we should have had smaller class sizes or extra staff to keep students apart. Its very unrealistic. Young children are impulsive and need the touch. They wrestle, hug, jump on each other. It is February and with daily frequent reminders they still have a hard time distancing. I worry that if 1 student got covid in my class, the likelihood that all of them would catch it is very high. We constantly do our best and cross our fingers! |  |
|  | Feeling criticized by public | 12 | - I am disgusted by the professional disrespect towards teachers. We truly have been getting the shitty end of the stick and we are all burning out. - In a year where teachers have made the most sacrifices, public/parent opinion feels to be overwhelmingly negative. It is extremely disheartening to me when I work so hard to create a community/meaningful learning opportunities. - The government and the public constantly bash teachers in the press and misrepresent us. The message is that us teachers are lazy, whiny, and not doing enough. Meanwhile, I am working more than ever before, giving 12-14 hours a day to my job, and experiencing adverse physical and mental health effects. Teachers are not allowed to speak publicly and defend ourselves, so we just keep getting beat down. I have never felt so depressed at my job before. | - M, 11, elementary - F, 4, secondary - F, 3, secondary |
|  | Feeling devalued and expendable | 22 | I feel really undervalued and disrespected by our government and the Ministry of Ed. I feel they are really putting my family at risk. We’ve done everything we can as a family to help bend the curve. Over night our contacts went from a tiny number to a massive number when school started. My wife and I both teach Gr.1/2 and have a teen in middle school and teen in high school. Our students aren’t wearing masks yet we wear ours all day. Our kids report that many kids aren’t wearing them in there classes. To me this is very stressful. It’s absolutely crazy that we don’t have a mask mandate and now we have even more contagious variants. The way this whole thing has been handled really makes me question staying in the profession. I never felt more disposable, like my family and myself don’t matter. It’s heartbreaking because I love working with kids and I know they love having me as a teacher. The whole “Be calm, be kind, be safe” mantra burns in my ears because it’s not kind to put some one in an unsafe situation and tell them to be calm. Do better, BC Gov. | M, 20, elementary |
|  | Feeling ostracized from community | 6 | Being a teacher and caregiver to young children has been unmanageable. My support system has become nonexistent due to concerns about the nature of my children in school and my work. I do my best to follow all guidelines but I watch students and parents around me not adhering to existing guidelines and it makes me feel unsafe. My personal stress level has never been this high. I was almost turned away for dental services due to the nature of my work. | F, 2, elementary |
|  | Increased workload | 34 | - As a DL teacher and district coordinator, my work load has often felt unmanageable and has caused a great deal of stress as it’s been impossible for me to keep up. I have felt like I’ve been in emergency mode since March and am now struggling with anxiety and terrible insomnia. - I definitely feel more stretched and fatigued this year. I’m working longer days too. | - F, 33, secondary - F, 21, elementary |
|  | Considering leaving profession | 26 | - This year has been very demanding and has solidified my decision to retire in June. - I contracted Covid and I feel very angry that I was put in this position at work. I am extremely frustrated and angry and I feel that I have no agency and no ability to protect myself and my family. I have never felt more undervalued and unappreciated in my career. I have considered quitting my job. | - F, 34, elementary - F, 7, secondary |
|  | More difficult to do job | 65 | If a pandemic can't get class sizes down to acceptable sizes, nothing will. It's a constant struggle/fight to get basic classroom needs met at the best of times. This year it has been a choice between trying to educate the kids for their academic needs OR try to keep them following COVID rules, both can't be done at the same time, they are both full-time jobs, so whatever you choose to do, there will be loss on either physical/social needs or academic needs. It feel like we are glorified babysitters, just trying to survive each day. There is so much interruption throughout the school year with COVID exposures and having to isolate, it's a joke we are even having to do report cards | F, 7, elementary/middle |
|  | Overwhelmed by changes | 8 | I do not think the public or ministry understand the constant change that staff are experiencing as schools adjust staffing levels to meet changing TL and onsite student numbers. I know teachers who are on their third assignment this year. It is exhausting to that teacher and to all teachers in the building to continuously replan/recommunicate/start over again and to be uncertain about the duration. On top of that there is a complete lack of resources for teachers to access. Planning is continuous and time consuming. Teachers have little energy left-over. Yet the ministry and district have not acknowledged this, have not reduced workload in other areas, and seem completely disconnected from the consequences of their decisions on teachers. It is only my reservoir of experience that gets me through. I can't imagine being a new or younger teacher at this time. | F, 30, elementary |
|  | Feeling disconnected from colleagues | 15 | - It’s been very isolating from colleagues, and this has a negative impact on us socially and professionally. - In the past we have been a very collaborative staff. Now people are just associating within small groups and there is a sense of disconnect and almost apathy. Everyone gets going home quite quickly after school. There is a heaviness even among the laughter. | - F, 20, elementary - F, 21, elementary |
| Concerns re: pandemic response | Feeling unsupported by government | 59 | - Teachers are being asked to teach as if we are in a normal setting with regular expectations with no recognition by the District nor Ministry if Education that we are in a pandemic and the priority it to keep everyone safe and healthy. - It has been a huge challenge with the lack of safety measures in schools to feel respected, supported, and valued by our government and the Ministry of Education. I feel expendable as a teacher in order to keep schools open to aid the economy. Teachers and school staffs are not given what is needed in this province at the best of times but especially now during a pandemic. My school district has done the best they can within the rules and mandates of this government re: protocols and regulations due to covid. I appreciate my colleagues who are hard working, dedicated and supportive during these challenging times. It has been a tough year in schools for staff and students. | - F, unknown, elementary - F, 38, elementary |
|  | Inconsistency in public health orders and implementation | 62 | The lack of transparency and inconsistent implementation and interpretation of PHO is a source of constant stress. Especially when the public and school orders do not match. I’m really upset when the statement is said that there is no data that supports school transmission when the data is not being collected or accurately collected. I have friends who are tracking nurses in different health authorities and their instructions, questions and follow up are so different. Of course there is no data, you are not collecting it or asking about it. The questions being asked do not take into account the school structures of adults that cross cohorts as a part of their job. | F, 16, middle |
| Student-related feedback | Missing connection with students and families | 11 | I miss close connections with my young students. | F, 20, elementary |
|  | Reduced learning time | 9 | Because we spend almost an hour and a half a day washing hands (30s/kid), we have significantly less instructional time, yet are still expected to teach the same material. I wished that the ministry sent out a statement telling the province to expect less curriculum this year. Furthermore, we still have to do the FSA exams further eating into our valuable instructional time and stressing kids and families out further when everyone is already at capacity | F, 15, elementary |
|  | Unable to meet students’ needs | 37 | - There are far more students struggling with anxiety and mental health and we’re struggling to provide enough support which creates even more stress and anxiety for teachers because we feel we are not supporting our students to the best of our ability. - We are all finding students to be quite low academically this year and finding it hard to meet their needs with our reduced schedule, especially their emotional needs as we have to keep our distance. | - F, 10, elementary - F, 24, elementary |
|  | Glad students are in school | 5 | - I am happy that schools are open for the kids. - Happy schools remained open, kids being in school is way more of a priority, than exposure to covid. The spread in my school has been very minimal. | - F, 17, secondary |
| Note. Total responses = 586/1276 | | | | |
